# Interoception and dissociation in migraine: a case–control study of chronic and episodic subtypes

**DOI:** 10.3389/fneur.2025.1643260

**Published:** 2025-08-20

**Authors:** Akihiro Koreki, Vilomi Bhatia, Anne-Marie Logan, Usman Khan, Mitsumoto Onaya, Sarah Garfinkel, Hugo Critchley, Mark Edwards, Niranjanan Nirmalananthan, Mahinda Yogarajah

**Affiliations:** ^1^Department of Clinical and Experimental Epilepsy, Institute of Neurology, University College London, London, United Kingdom; ^2^Department of Psychiatry, NHO Shimofusa Psychiatric Medical Center, Chiba, Japan; ^3^Institute of Cognitive Neuroscience, University College London, London, United Kingdom; ^4^Atkinson Morley Regional Neuroscience Centre, St George’s Hospital, London, United Kingdom; ^5^Department of Clinical Neuroscience, Brighton and Sussex Medical School, Sussex University, Sussex, United Kingdom; ^6^Neurology and Interface Disorders at the Institute of Psychiatry, Psychology and Neuroscience at King’s College London, London, United Kingdom; ^7^Chalfont Centre for Epilepsy, NIHR University College London Hospitals Biomedical Research Centre, London, United Kingdom

**Keywords:** chronic, dissociation, interoception, migraine, predictive coding

## Abstract

**Background:**

Migraine is one of the most common neurological disorders. Despite advances in understanding of episodic migraine, little is understood about the mechanisms underlying the chronification of migraine. Recently, increasing attention has been given to the potential roles of interoceptive abnormalities and dissociation. Therefore, we sought to explore differences in interoception and dissociation in individuals with episodic and chronic migraine versus individuals without migraine.

**Methods:**

A total of 49 participants were analysed of which 26 had migraine (15 chronic and 11 episodic) and 23 were control subjects without a headache disorder. Their objective interoceptive accuracy was assessed using the heartbeat tracking and discrimination tasks. Interoceptive sensibility was assessed using the Porges body perception questionnaire. Interoceptive trait prediction error (ITPE) was calculated based on the discrepancy between their task performance and sensibility. Interoceptive state prediction error (ISPE) was calculated based on the trial-by-trial correspondence between task performance and confidence. The level of their dissociation was assessed via self-report questionnaires.

**Results:**

Patients with migraine had lower interoceptive accuracy for the tracking task (median (interquartile range) 0.50 (0.43) in migraine vs. 0.78 (0.26) in control, Mann–Whitney U test, effect size r = 0.35, *p* = 0.014), higher interoceptive sensibility (110 (52) vs. 39 (14), r = 0.74, *p* < 0.001), and greater ITPE than controls (for the tracking task: 1.08 (1.78) vs. − 1.16 (0.88), r = 0.72, *p* < 0.001 / for the discrimination task: 0.87 (1.44) vs. − 0.62 (0.97), r = 0.69, *p* < 0.001). Greater ISPE was also found in patients with chronic migraine than episodic migraine (2.30 (0.35) in chronic vs. 1.75 (0.19) in episodic, r = 0.39, *p* = 0.046). A greater level of somatoform dissociation was found in individuals with chronic, compared to episodic, migraine (27 (11) vs. 22 (2), r = 0.43, *p* = 0.029).

**Conclusion:**

This is the first study to demonstrate interoceptive abnormalities in migraine, specifically of greater interoceptive prediction errors. Interoceptive abnormalities may represent a transdiagnostic mechanisms relevant to the chronification of migraine, and to frequent co-morbidities such as dissociation.

## Graphical abstract

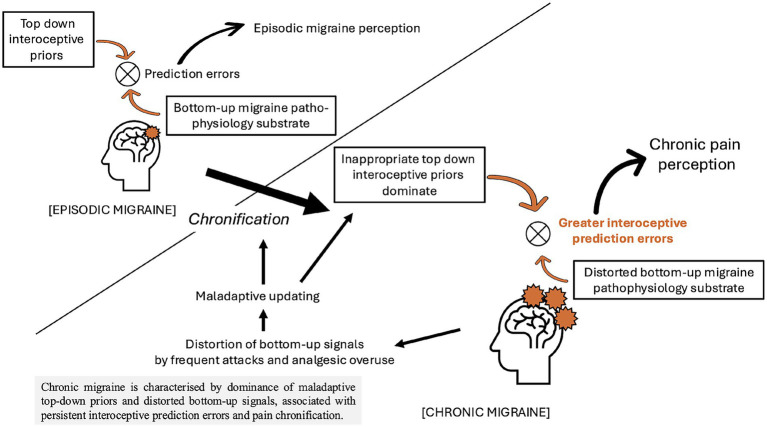

## Introduction

Migraine is a complex and common episodic neurological condition characterised by a combination of exteroceptive (symptoms resulting from sensory perception from the world external to the body such as photo- or phono-sensitivity) and interoceptive phenomena (symptoms resulting from sensory perception from the body itself such as premonitory auras). The impact of migraine extends beyond the excruciating pain and discomfort experienced during migraine attacks, influencing various aspects of an individual’s life, including emotional well-being, quality of life, and cognitive functioning (1). While research into migraine biology and management has made considerable advances, understanding of those individuals with chronic migraines and migraine co-morbidities remains limited (2, 3).

Interoception refers collectively to the body-to-brain axis of signals originating from the internal body and visceral organs, and encompasses the predictive representation and control of one’s physiological state—the state of “minimal selfhood” (4). Interoception, therefore, provides insights into how the body’s internal state and sensations are perceived and regulated. Changes in the body’s internal state trigger automatic physiological reactions and mental sensations like hunger, thirst, pain, and emotions. There is clinical and neuroimaging evidence that supports the involvement of interoceptive systems in migraine (5, 6). During the prodromal phase individuals with migraine may experience specific interoceptive feelings (e.g., fatigue, hunger) (7), while the headache phase is characterised by other interoceptive phenomena such as pain (8). Functional neuroimaging studies highlight the important role played by brain structures such as the brain stem, insula, and default mode network in migraine, which are also all important interoceptive hubs (1). Finally, discrepancies between objective and subjective aspects of interoception underpin the integrity of self-representation, a cornerstone of dissociation (9). We have previously demonstrated the relevance of this relationship in patients with functional seizures (10). Many individuals with migraine also suffer from functional neurological disorders and dissociative symptoms (11), including both psychoform (disconnection from one’s thoughts, emotions, or surroundings) and somatoform (disconnection from one’s body) dissociation (12, 13).

Given the frequent co-morbidity of functional seizures, dissociation, and migraine and the interoceptive abnormalities identified in other chronic pain disorders such as fibromyalgia and chronic low back pain (8, 11), we hypothesised that patients with migraine would have interoceptive abnormalities, and that these may correspond to levels of dissociation. While migraine is typically an episodic neurological disorder, dissociation has been shown to play an important role in the development of chronic migraine (14, 15). We therefore also hypothesised that there may be dissociative and interoceptive differences between individuals with chronic and episodic migraine. In this study, we sought to explore interoception and dissociation using cardiac interoceptive paradigms and self-report questionnaires.

## Methods

### Design and participants

This was a case–control study that locally recruited a total of 50 participants. After excluding one patient due to a lack of task performance data on our main outcome, due to technical failure, we finally analysed a total of 49 participants, of which 26 had migraine (15 chronic and 11 episodic) and 23 were individuals without headaches, matched for age and sex and recruited from staff and students at the study institution. Educational and socio-economic data were not available for control individuals. Patients over 18 years old who presented for headache symptoms at neurology clinics at the Atkinson Morley Regional Neuroscience Unit at St. George’s University Hospital, London, from October 2019 to February 2020, were screened for eligibility using the International Classification of Headache Disorders, 3rd edition (ICHD-3) diagnostic criteria by suitably qualified headache clinicians (16). Patients were classified as suffering either episodic migraine (<15 days a month of headache) or chronic migraine (≥15 days a month of headache of which ≥8 were migraine) based on these criteria. Patients that were pregnant, on medications with direct cardiac effects, such as beta-blockers, or had severe language or learning disabilities were excluded from the study. We confirmed that no participants experienced a migraine attack during their assessments. The participants were patients who had been newly referred by their general practitioners to a neurology headache clinic in our centre. For this reason, the vast majority of patients were not taking regular preventative medications for their headaches. One patient was taking topiramate, and another had recently received a greater occipital nerve block in the emergency department. Most patients were using over-the-counter abortive medications (e.g., paracetamol, ibuprofen, triptans). Six participants had significant comorbid conditions which included functional neurological disorder (chronic patient), anxiety disorder (one episodic and chronic patient), depression (one episodic and chronic patient), and post-traumatic stress disorder (chronic patient). No patients met the criteria for vestibular migraine. The ethics committee of Fulham, London approved the study protocol, as did the Health Research Authority (IRAS ID: 231863). The study was conducted in accordance with the ethical guidelines set forth by the Declaration of Helsinki. All participants provided written informed consent.

## Materials

Interoceptive function was assessed using a dimensional framework which included several self-report questionnaires, and cardiac interoceptive tests. The experiment was conducted in a quiet room with no way of telling the time. Participants were sat upright facing away from the experimenter and wore a finger pulse oximeter (Nonin Xpod 3012LP) connected to a laptop, providing an objective measurement of heart rate. The finger pulse oximeter method has been validated against electrocardiogram (ECG) for heartbeat timing (17, 18).

### A heartbeat tracking task (HTT) and a heartbeat discrimination task (HDT)

In the HTT, participants were asked to count their own heartbeats, without touching any body part, during six-time windows (randomized trials of 25, 30, 35, 40, 45 and 50 s.), and to report the number of heartbeats detected at the end of each trial. Interoceptive accuracy for the HTT was calculated on a trial-by-trial basis as below: 1 – (|nbeats_real_ – nbeats_reported_|)/((nbeats_real_ + nbeats_reported_)/2), and these were averaged to form a mean score (10, 19).

In the HDT, participants were asked to listen to ten tones (440 Hz) in each trial and then report their judgement whether the tones were in synchrony with their own heartbeat or not (10, 19). There were 20 trials in total. As the synchronous condition, tones were triggered by onset of the finger pulse waveform (i.e., on average ~250 ms after the R-wave). As the out-of-synch (delayed) condition, tones were presented at ~550 ms after the R-wave. These conditions correspond to maximum and minimum synchronicity judgements, respectively (20). Interoceptive accuracy for the HDT was calculated as a ratio of correct to incorrect judgments.

As a control task for the HTT, a time tracking task was conducted. They are asked to report the number of seconds of the six randomized various time windows, following the same procedure as in the HTT. Time accuracy was also calculated in the similar way to the HTT.

### Interoceptive sensibility

Interoceptive sensibility is patients’ subjective belief concerning interoceptive sensations and experiences assessed using questionnaires (21). In the present study their sensibility was quantified by Porges Body Perception Questionnaire Awareness Scale (BPQ-A) (22).

### Interoceptive prediction error

Conceptually based on predictive coding models extended to interoception (23, 24), the interoceptive prediction error is a gap between subjective belief and objective performance. Two kinds of interoceptive prediction error have been proposed: interoceptive trait prediction error (ITPE) and interoceptive state prediction error (ISPE) (10). ITPE is a gap between interoceptive sensibility (subjective belief) and interoceptive accuracy (objective performance) (21). The scores of interoceptive sensibility and accuracy for the HTT and HDT were converted to standardized Z-scores in whole group, and ITPE for the HTT and HDT was calculated based on subtraction from sensibility to accuracy, using the formula: ITPE = Z-score (interoceptive sensibility) − Z-score (interoceptive accuracy). Since Z-scores were used, this value reflects individual propensity relative to the entire sample. A higher, positive ITPE value indicates a tendency to overestimate interoceptive ability, characterized by a stronger subjective belief in one’s ability despite poor objective performance. Conversely, a lower value is reflected in a more subdued subjective belief accompanied by better objective performance (21). In contrast, ISPE represents the trial-by-trial correspondence between task performance and confidence, and can be assessed based on the performance and confidence in the HDT. In the HDT, they were asked to report their confidence in judgment using a visual analogue scale ranging from total guess to complete confidence in each trial. To calculate ISPE, metacognitive interoceptive awareness was first calculated as area under the curve of a receiver operating characteristic (ROC) analysis, which was based on trial-by-trial task performance (correct or incorrect) and trial-by-trial confidence ratings (numerical values) in the HDT. ISPE was the inverse of metacognitive interoceptive awareness. Positive values of these error values indicate a propensity for individuals to overestimate their subjective relative to their objective cardiac interoceptive perceptual ability among our sample.

### Assessment of dissociation, anxiety and depression and severity of migraine

In both patient and control groups, dissociation was assessed using the somatoform dissociation questionnaire (SDQ) (25) and the multiscale dissociation inventory (MDI) (26). Anxiety and depression were assessed using the Beck’s anxiety inventory (BAI) (27) and Beck’s depression inventory (BDI) (28). The presence of PTSD and/or a trauma history was not assessed due to ethical constraints. In patients only, symptom severity was assessed using migraine disability assessment (MIDAS) (29) and headache impact test (HIT) (30).

### Statistics

Due to small sample size and non-parametrically distributed data, their performance and the scores were compared between patients with migraine and the non-clinical sample using the Mann–Whitney U test. Group differences in gender were compared using the Fisher’s exact test. To demonstrate the difference between patients with chronic and episodic migraine, comparison between clinical subtypes was also conducted in the same manner. Missing values accounted for 3.1% of the entire dataset, predominantly in questionnaire-based variables. To address missingness, we applied multiple imputation by chained equations, including all variables used in the analyses. We generated 20 imputed datasets, each created through a maximum of 50 iterations to ensure convergence. The results were pooled for inference following Rubin’s rules (31). For graphical representation, each participant’s median value across imputations was plotted. To clarify group differences, general linear models (GLMs) were conducted when ITPE/ISPE significantly differed between groups, with interoceptive prediction errors as the dependent variables, and groups and potential confounders that differed between groups as the independent variables. The Spearman correlation analyses were conducted between interoception-related parameters and their symptoms. The effect size (r-value in the Mann–Whitney U test, phi-value in the Fisher’s exact test, rho-value in the Spearman correlation analysis) was calculated, and was interpreted as small, medium or large, with values around 0.10 considered small, around 0.30 medium, and 0.50 or above large (32). The threshold of 0.05 was used to consider significance. This was a hypothesis generating study and for this reason we explicitly report significant results without multiple comparison correction. However, for the sake of transparency and completeness, we also report results with multiple comparison correction. The false discovery rate (FDR) using the Benjamini and Hochberg method was used for controlling multiple statistical testing (33). In addition, post-hoc power analyses were conducted for the main group comparisons to evaluate the statistical sensitivity and potential power limitations of the study. Statistical analyses were carried out using R (4.3.2).

## Results

### Comparison between patients with migraine and non-clinical samples

No significant difference was found for age [median (interquartile range: IQR)]: 31 (21) in migraine and 27 (10) in non-clinical samples, *p* = 0.197 and sex (male/female: 9/17 in migraine and 5/18 in non-clinical samples, *p* = 0.360). Significantly greater levels of anxiety (*p* = 0.049) and depression (*p* < 0.001) were found in patients compared to non-clinical samples. A greater level of dissociation including somatoform dissociation was found (*p* < 0.001 in SDQ and 0.006 in MDI total score) in patients than non-clinical samples (Table 1). Regarding the subscales of MDI, depersonalization (*p* < 0.001), derealization (*p* = 0.007), memory disturbance (*p* = 0.007), and identity dissociation (*p* < 0.001) were significant. Poorer interoceptive accuracy measured by the HTT was found in patients with migraine compared to non-clinical samples (0.50 (0.43) in migraine and 0.78 (0.26) in non-clinical samples, *p* = 0.014), while there were no differences of interoceptive accuracy measured by the HDT and time accuracy. Greater interoceptive sensibility in patients with migraine than non-clinical samples assessed by BPQ-A was found (110 (52) and 39 (14) respectively, *p* < 0.001). The significance of group differences persisted even after FDR corrections, except for anxiety. In addition, the effect sizes observed in these group differences were considered to be of medium to large size (Table 1).

**Table 1:** Comparison between patients with migraine and control.

	Control (*n* = 23)	Migraine (*n* = 26)	Effect size	*p*-value
General information				
Age, median (IQR)	27 (10)	31 (21)	0.18	0.197
Sex, M/F	5/18	9/17	0.32	0.360
Anxiety & depression				
BAI, median (IQR)	5 (5)	9 (26)	0.28	**0.049**
BDI, median (IQR)	1 (4)	9 (16)	0.57	**<0.001**
Dissociation				
SDQ, median (IQR)	20 (2)	23 (7)	0.56	**<0.001**
MDI, median (IQR)	36 (6)	45 (18)	0.39	**0.006**
Tasks & interoceptive sensibility				
HTT, median (IQR)	0.78 (0.26)	0.50 (0.43)	0.35	**0.014**
HDT, median (IQR)	0.50 (0.20)	0.48 (0.20)	0.24	0.098
TTT, median (IQR)	0.78 (0.23)	0.77 (0.16)	0.00	0.976
Interoceptive sensibility, median (IQR)	39 (14)	110 (52)	0.74	**<0.001**

Rows shaded in grey and *p*-values shown in bold indicate statistical significance. BAI: Beck’s Anxiety Inventory; BDI: Beck’s Depression Inventory; HDT: Heartbeat Discrimination Task; HTT: Heartbeat Tracking Task; IQR: Interquartile Range; ISPE: Interoceptive State Prediction Error; ITPE: Interoceptive Trait Prediction Error; MDI: Multiscale Dissociation Inventory; SDQ: Somatoform Dissociation Questionnaire; TTT: Time Tracking Task.

**Figure 1:**
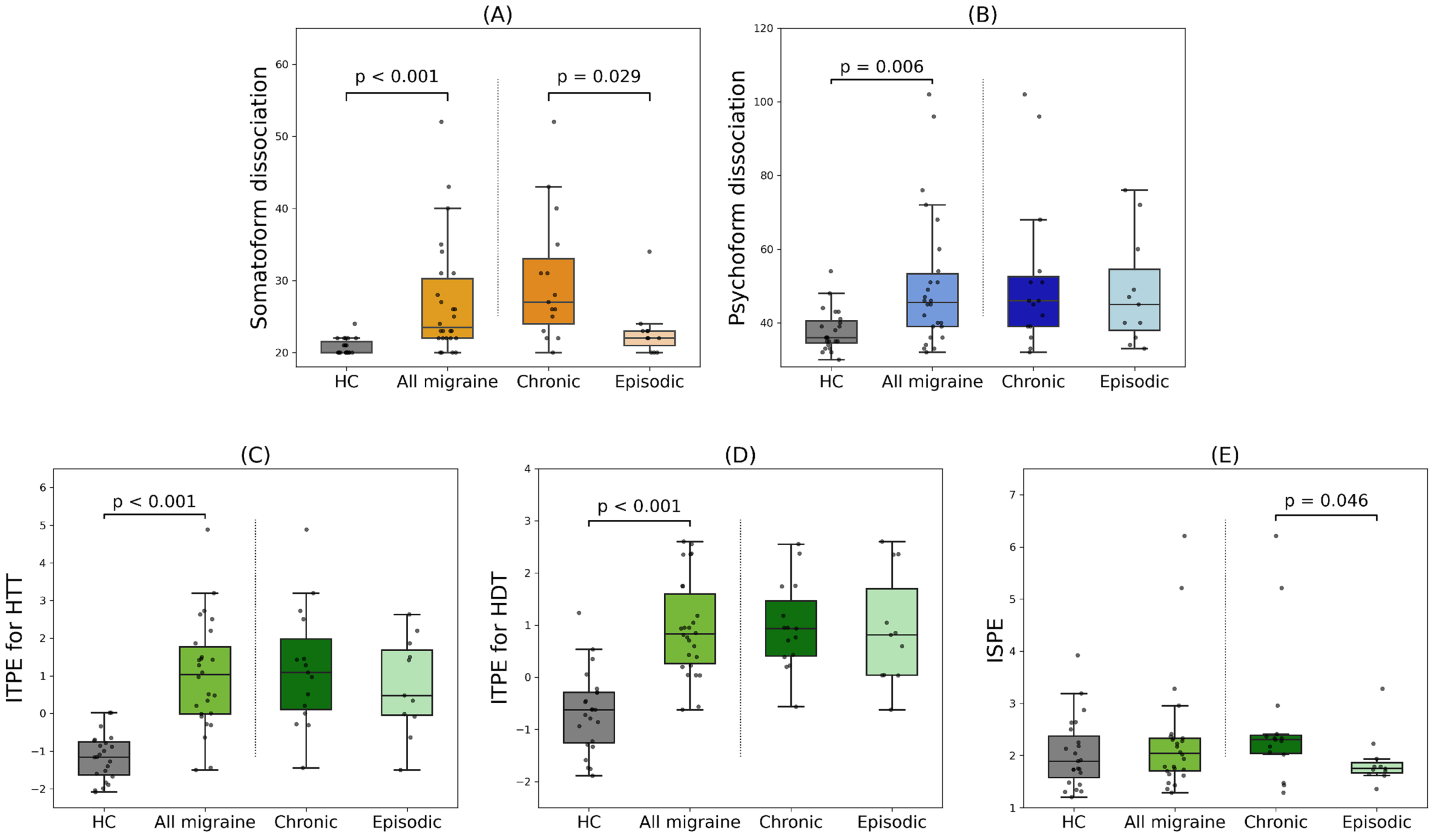
**(A,B)** Greater levels of somatoform and psychoform dissociation were found in patients with migraine than healthy control. In addition, a more severe level of somatoform dissociation was found in patients with chronic migraine than episodic migraine. **(C,D)** Greater levels of interoceptive prediction error for the heartbeat tracking task (HTT) and heartbeat discrimination task (HDT) were found in patients with migraine than in healthy control. **(E)** A significantly greater introceptive state prediction error was found in patients with chronic migraine than episodic migraine. HC: healthy control, ITPE: interoceptive trait prediction error, ISPE: interoceptive state prediction error.

Greater ITPEs for the HTT and HDT were found in migraine (1.08 (1.78) and 0.87 (1.44), respectively) than in non-clinical samples [−1.16 (0.88) and −0.62 (0.97) (both <0.001)]. No difference in ISPE was found between groups (Table 2; Figure 1). In the GLM that included SDQ, MDI, BAI, and BDI scores, which significantly differed between groups, the group difference in ITPE for the HTT remained significant (*β* = 1.12, *p* < 0.001) even after controlling for potential confounders; SDQ (*p* = 0.332), MDI (*p* = 0.641), BAI (*p* = 0.966) and BDI (*p* = 0.956). Similarly, the GLM for ITPE for the HDT revealed significant group difference (β = 1.20, *p* < 0.001) even after controlling potential confounders; SDQ (*p* = 0.387), MDI (*p* = 0.595), BAI (*p* = 0.153) and BDI (*p* = 0.101) (Table 2). Post-hoc power analyses indicated a power of 0.996 for ITPE for the HTT and 0.999 for ITPE for the HDT.

**Table 2:** Comparison of interoceptive prediction errors between patients with migraine and control.

	Control (*n* = 23)	Migraine (*n* = 26)		Control vs. Migraine		
			Univariable		Multivariable^†^	
			Effect size (r)	*p*-value	β	*p*-value
ITPE for HTT, median (IQR)	−1.16 (0.88)	1.08 (1.78)	0.72	**<0.001**	1.12	**<0.001**
ITPE for HDT, median (IQR)	−0.62 (0.97)	0.87 (1.44)	0.69	**<0.001**	1.20	**<0.001**
ISPE, median (IQR)	1.89 (0.80)	2.04 (0.63)	0.10	0.483	-	-

Rows shaded in grey and *p*-values shown in bold indicate statistical significance. HTT: Heartbeat Tracking Task, HDT: Heartbeat Discrimination Task, IQR: Interquartile range, ISPE: Interoceptive state prediction error, ITPE: Interoceptive trait prediction error. †: This model includes potential confounding factors in group differences, namely the scores of the Somatoform Dissociation Questionnaire, Multiscale Dissociation Inventory, Beck Anxiety Inventory, and Beck Depression Inventory.

### Comparison among clinical migraine types

Among migraine patients, individuals with chronic migraine were significantly older than those with episodic migraine (38 (14) and 22 (2) respectively, *p* = 0.001), while no significant difference for sex (male/female: 6/9 in chronic and 3/8 in episodic, *p* = 0.683) was found. A more severe level of somatoform dissociation (*p* = 0.029) was found in chronic migraine. No differences in interoceptive accuracy measured either by the HTT or HDT, time accuracy, and interoceptive sensibility were found (Table 3). The significance of group differences did not survive after FDR corrections, mainly due to a small sample size, but the effect sizes observed in these group differences were medium to large.

**Table 3:** Comparison between patients with chronic and episodic migraine.

	Chronic (*n* = 15)	Episodic (*n* = 11)	effect size	*p*-value
General information				
Age, median (IQR)	38 (14)	22 (2)	0.63	**0.001**
Sex, M/F	6 /9	3 / 8	0.28	0.683
Anxiety & depression				
BAI, median (IQR)	15 (26)	7 (17)	0.18	0.350
BDI, median (IQR)	11 (23)	6 (6)	0.22	0.272
Dissociation				
SDQ, median (IQR)	27 (11)	22 (2)	0.43	**0.029**
MDI-total score, median (IQR)	45 (19)	45 (17)	0.01	0.960
Tasks & interoceptive sensibility				
HTT, median (IQR)	0.53 (0.41)	0.48 (0.42)	0.06	0.775
HDT, median (IQR)	0.45 (0.20)	0.50 (0.20)	0.08	0.697
TTT, median (IQR)	0.74 (0.16)	0.77 (0.13)	0.06	0.770
Interoceptive sensibility, median (IQR)	112 (46)	104 (55)	0.13	0.505
Severity of headache				
Monthly headache frequency, median (IQR)	30 (8)	2 (3)	0.71	**<0.001**
MIDAS, median (IQR)	25 (50)	7 (13)	0.24	0.218
HIT, median (IQR)	65 (11)	56 (11)	0.33	0.088

Rows shaded in grey and *p*-values shown in bold indicate statistical significance. BAI: Beck’s Anxiety Inventory; BDI: Beck’s Depression Inventory; HDT: Heartbeat Discrimination Task; HIT: Headache Impact Test; HTT: Heartbeat Tracking Task; IQR: Interquartile Range; ISPE: Interoceptive State Prediction Error; ITPE: Interoceptive Trait Prediction Error; MDI: Multiscale Dissociation Inventory; MIDAS: Migraine Disability Assessment Scale; SDQ: Somatoform Dissociation Questionnaire; TTT: Time Tracking Task.

Greater ITPE was found in chronic migraine, but did not reach a significance due to small sample size (Table 4). Greater ISPE was found in chronic than episodic migraine (2.30 (0.35) in migraine and 1.75 (0.19), *p* = 0.046) (Table 4; Figure 1). The GLM for ISPE revealed a significant difference between clinical migraine types (*β* = 1.11, *p* = 0.042) even after controlling potential confounders; Age (*p* = 0.434) and SDQ (*p* = 0.193) (Table 4). A post-hoc power analysis indicated a power of 0.584 for ISPE.

**Table 4:** Comparison of interoceptive prediction errors between patients with chronic and episodic migraine.

	Migraine			Chronic vs. Episodic		
	Chronic (*n* = 15)	Episodic (*n* = 11)	Univariable		Multivariable^‡^	
			Effect size (r)	*p*-value	β	*p*-value
ITPE for HTT, median (IQR)	1.15 (1.82)	0.48 (1.73)	0.12	0.530	-	-
ITPE for HDT, median (IQR)	0.94 (1.18)	0.81 (1.66)	0.10	0.614	-	-
ISPE, median (IQR)	2.30 (0.35)	1.75 (0.19)	0.39	**0.046**	1.11	**0.042**

Rows shaded in grey and *p*-values shown in bold indicate statistical significance. HTT: Heartbeat Tracking Task, HDT: Heartbeat Discrimination Task, IQR: Interquartile range, ISPE: Interoceptive state prediction error, ITPE: Interoceptive trait prediction error. ‡: This model includes age and the scores of Somatoform Dissociation Questionnaire.

### Association between dissociation and interoceptive prediction errors

**Figure 2:**
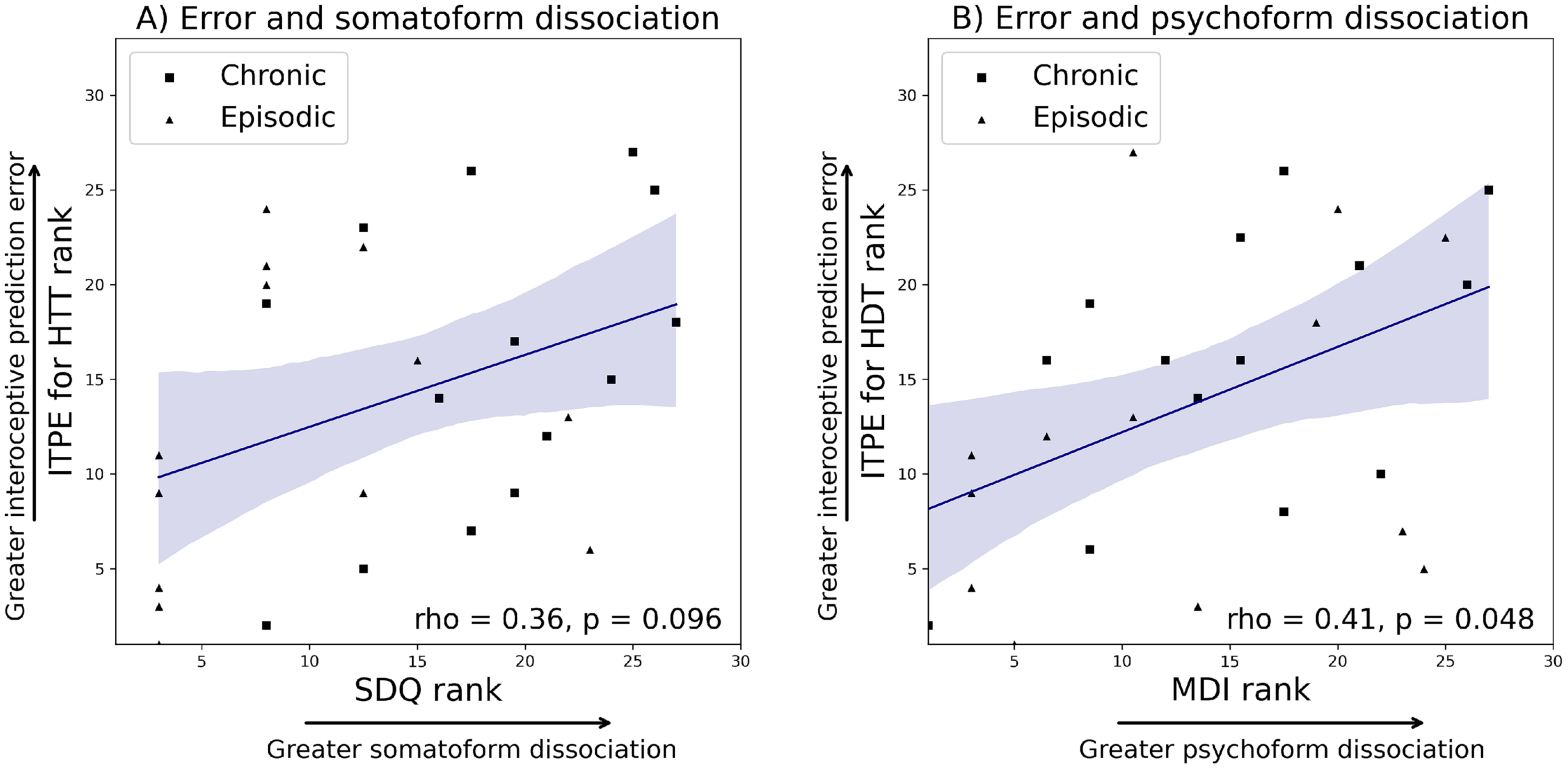
Ranks were plotted to ensure consistency with Spearman correlation. **(A)** Correlation analyses across all migraine patients revealed that SDQ had a trend-level association with ITPE for the heartbeat tracking task (rho = 0.36, *p* = 0.096). **(B)** MDI total score had a significant association with ITPE for the heartbeat discrimination task (rho = 0.41, *p* = 0.048). ITPE: interoceptive trait prediction error, ISPE: interoceptive state prediction error. MDI: multiscale dissociation inventory, SDQ: somatoform dissociation questionnaire.

Correlation analyses across all migraine patients revealed a significant association between the MDI total score and ITPE for the HDT (rho = 0.41, *p* = 0.048), and a trend-level association between the SDQ and ITPE for the HTT (rho = 0.36, *p* = 0.096). No association was found between the SDQ and ITPE for the HDT (*p* = 0.234) or ISPE (*p* = 0.738). Similarly, the MDI total score was not associated with ITPE for the HTT (*p* = 0.189) or ISPE (*p* = 0.613). The observed significance between MDI and ITPE for the HDT did not survive after FDR corrections, mainly due to a small sample size, but the observed effect sizes, expressed as correlation coefficients, were in the medium range (Figure 2).

## Discussion

In this study we have identified for the first time abnormalities of interoception in individuals with migraine with a moderate to large effect size. These individuals had poorer interoceptive accuracy as indexed by the HTT, greater interoceptive sensibility, and greater ITPE in comparison to individuals who did not suffer from migraines. Greater levels of dissociation including psychoform and somatoform dissociation, as well as anxiety and depression, were also found in individuals with migraine. However, differences were also identified between individuals with episodic and chronic migraine. Greater somatoform dissociation was found in individuals with chronic versus episodic migraine. These findings in relation to dissociation are broadly in keeping with the published literature (13, 34). A few studies have investigated differences in dissociation between individuals with chronic and episodic migraine and have typically focused on psychoform dissociation, which is noted to be elevated, and an important mediator of the development of chronic migraines (15, 35). Importantly, greater ISPE and greater ITPE for the HTT and HDT were identified in individuals with chronic migraine, although only in the former case was this significant. Our GLMs highlight significant differences in ITPE between migraine and control groups and in ISPE between clinical migraine subtypes, while allowing for confounding variables. These findings overlap with those reported in patients with FMD and FS (10, 36), and may therefore be relevant to our mechanistic understanding of why migraines and functional movement disorders/functional seizures are frequently co-morbid (37, 38). Our findings of a correlation between dissociation and ITPE across all individuals with migraine are also in keeping with this finding. The overall task performances and questionnaire scores of healthy controls in the current study are similar to those in our previous study (10), supporting the validity of our current interoception and dissociation assessment methods, and emphasising the value of these findings.

The interoceptive findings in this study can be understood through a framework of predictive processing, given that subjective experience, including pain perception, has been recently re-conceptualised from the viewpoint of these frameworks (39). Historically, models of subjective experience were based on the idea that they emerge from bottom-up processing of sensory signals by the brain. In contrast, in predictive processing models, subjective experience emerges as perceptual inference based on the discrepancy between bottom-up sensory signals and top-down predictions (priors), and the process to minimise this discrepancy. The model has been used to explain exteroceptive modalities, such as vision, but has recently been extended to interoception (23, 24). In an extended interoceptive model, the sense of body ownership is determined by the minimization of the discrepancy between top-down predictions about the interoceptive state of the body and bottom-up incoming interoceptive signals evoked (directly) by autonomic control signals and (indirectly) by bodily responses to afferent sensory signals. Conversely, this model maintains that disorders of body ownership, such as dissociation, result from pathologically imprecise interoceptive predictive signals and increased discrepancy between top-down and bottom-up signals (9).

ITPE is a metric of the divergence between the subjective longer-term belief/self-model of general interoceptive ability and objective sensory sensitivity to interoceptive information. It can also be conceived as the discrepancy between the precision (weighting) afforded to subjective belief/self-model of general interoceptive ability, and the ability to adjust or update these predictions in the light of new bottom-up sensory information. Differences in ITPE between individuals with and without migraine may therefore reflect disordered bodily predictions by the brain. These may arise because the occurrence of migraine, regardless of whether it is episodic or chronic, contributes to increased levels of both the attention that an individual pays to their body, and their subjective perception of their accuracy in perceiving those bodily signals. However, there may be a lack of a corresponding increase in the objective accuracy of detection of bottom-up bodily signals due to structural changes in interoceptive networks in patients with migraine (1). Indeed, patients with migraine scored lower on objective tests of interoceptive accuracy and higher on interoceptive sensibility measures which index attention or weighting afforded to subjective bodily signal appraisal, compared to individuals without migraine. Furthermore, as we have demonstrated in previous work in patients with FS (10), there was a trend toward a correlation between ITPE scores and both psychoform and somatoform dissociation in individuals with migraine.

In contrast to ITPE, ISPE differed between migraine subgroups, and was greater in individuals with chronic migraine. ISPE represents the discrepancy between interoceptive accuracy and a participant’s trial-by-trial subjective opinion of their interoceptive accuracy. Prediction errors in this setting refer to state moment-to-moment discrepancies between expected and actual interoceptive signals, rather than trait-based differences between objective and subjective performance (as indexed by ITPE). Within predictive coding models, subjective bodily experiences arise from the discrepancy (i.e., interoceptive prediction error) between bottom-up interoceptive signals and top-down predictions of internal bodily states. This model proposes that adaptive, normal functioning relies on the relative precision (weighting) of error signals and predictions to adjust expectations and perceptions for any given context. In this model the persistence of imprecise error signals can lead to situations where highly precise but inaccurate priors or predictions (e.g., the presence of a headache in an individual who suffers with frequent headaches) may dominate the generative model for somatic stimuli, leading to perceived symptoms in individuals which are not a perfect correlate of bodily, physiological activity. This finding may therefore contribute to the development of central sensitization and chronic pain in individuals with chronic migraine, where the pain experienced by individuals is not in proportion to relevant bodily physiological activity, such as the activation of trigeminovascular neurons and subsequent inflammatory cascades, typically seen in episodic migraine (40). This mechanism may also be partly responsible for medication overuse being a risk factor in the transformation from episodic to chronic migraine and medication overuse headaches (MOH) (14). Here, frequent use of analgesics, including over-the-counter medications, will dampen bottom-up interoceptive signaling pathways (41), reducing opportunities for the brain to recalibrate priors in light of actual bodily states. This can result in an over-reliance on maladaptive top-down predictions of pain and a heightened sensitivity to interoceptive prediction errors when medication effects subside. Such a mechanism may perpetuate headache frequency and drive further analgesic use, culminating in MOH as a maladaptive allostatic response. In contrast, individuals who do not frequently use analgesics may avoid the maladaptive cycle underlying MOH. In such individuals, the nervous system continues to receive intact bottom-up interoceptive signals from nociceptive pathways, enabling accurate updating of top-down expectations and maintaining an appropriate precision balance between predictions and sensory input. The system remains better able to adapt to normal fluctuations in pain and avoids developing a self-reinforcing cycle of increased prediction error and increased top-down expectation of pain.

The findings in this study parallel our work in individuals with functional seizures where, ITPE predicted dissociation, and ITSE predicted seizure frequency significantly (10). These parallel findings may therefore reflect shared mechanisms underpinning the frequent co-occurrence of chronic migraines and functional seizures (11). Furthermore, this importance of interoceptive prediction errors in migraine is compatible with the recently proposed theory that migraine can be understood as an allostatic reset triggered by unresolved interoceptive prediction errors (5).

The inclusion of many individuals with very frequent or chronic daily headaches, all of whom met ICHD-3 criteria for chronic migraine, is an important feature of this study. While classical episodic migraine has well-characterised neurobiological mechanisms, patients with chronic and particularly daily headache often present with a more complex clinical profile that may not be fully explained by these models alone. Our data suggest that this subgroup is characterised by elevated dissociation and altered interoceptive prediction error, pointing to additional mechanisms involving self-representation and bodily awareness. These features overlap with those seen in functional neurological disorders and may reflect a broader set of transdiagnostic processes relevant to symptom chronification and central sensitisation. Rather than representing a source of diagnostic ambiguity, patients with daily headache may instead offer key insights into the multidimensional nature of migraine, particularly the transition from episodic to chronic forms. This supports the view that chronic migraine is not merely an extension of episodic migraine in frequency, but may involve qualitatively different underlying processes. As such, studying individuals with daily headache is essential for identifying mechanisms that contribute to migraine refractoriness, symptom amplification, and co-occurring functional symptoms. These findings may help to bridge biological and psychophysiological models of migraine and open new avenues for therapeutic intervention.

These findings have several potential clinical implications. First, they suggest that in patients with frequent or chronic migraine, especially those reporting dissociative experiences or high symptom burden not fully explained by classical mechanisms, clinicians might consider assessing aspects of interoceptive awareness or bodily self-perception. This could be as simple as including questions about how accurately patients perceive bodily signals or how distressed they are by them. Second, recognising altered interoceptive prediction errors as a possible contributor to symptom maintenance highlights a target for intervention. Emerging approaches such as interoceptive training, body-focused mindfulness, or even neuromodulatory techniques like vagus nerve stimulation might help recalibrate interoceptive processing, potentially reducing symptom amplification and preventing chronification. For example, non-invasive vagus nerve stimulation (VNS) represents a non-pharmacological treatment option for migraine. Interestingly, recent studies have revealed that VNS facilitates interoceptive processing and reduces an individual’s susceptibility to dissociation and disembodiment (42–44). The findings in this study suggest that one potential mechanism of action of VNS in migraine may be the modulation of interoceptive systems. VNS may therefore have a role in the prevention and treatment of chronic migraine and its comorbidities, such as dissociation. Finally, understanding that dissociation and altered bodily awareness may be intertwined with migraine could guide clinicians to screen for co-occurring functional neurological symptoms, inform psychoeducation, and foster a more integrated biopsychosocial treatment plan.

This is the first study to investigate interoception in individuals with episodic and chronic migraine, and in doing so, it highlights potential links to functional movement disorders/functional seizures. However, several limitations remain. First, the small sample size is a significant limitation. Although a statistically significant group difference in ISPE was observed, the post-hoc power for this comparison was relatively low indicating a limited ability to consistently detect this effect. Therefore, the findings in individuals with episodic and chronic migraine need to be replicated and extended with a broader spectrum of migraine patients. Second, although individuals taking medications with direct cardiac effects were excluded, some individuals were taking other medications, including as-required pain killers, and the effect of medication on our findings cannot be controlled. Since even simple over-the-counter analgesic medications may block bottom-up interoceptive signaling pathways and thereby alter prior beliefs and precision weighting, they should be considered when exploring the interoceptive mechanisms of migraine chronification. Further studies are therefore needed to clarify the influence of analgesic medication use on interoceptive processing. Third, while none of the participants included in the study experienced a migraine either during the assessments or earlier on the same day, no information was collected regarding the interval between their last (or subsequent) migraine attacks and the assessments. This makes it challenging to determine the direct influence of migraine attacks on our findings. Since headache attacks themselves may transiently alter interoceptive processing, further studies investigating the temporal dynamics of interoception following attacks are warranted. Fourth, knowledge and beliefs regarding one’s own heart rate may affect the HTT results, making it less representative as a measure of true interoception (45). In order to address this, we included the time tracking task as a control task. However, future studies should consider using other interoceptive tasks, such as heartbeat-evoked potentials or tasks assessing cardiac modulation of cognition, which may provide more implicit and objective indices (46, 47). Fifth, we acknowledge that certain potential confounding factors, such as educational level and socioeconomic status, were not collected in this study. These variables may plausibly influence interoceptive functioning, and this limitation should be considered when interpreting the findings. Sixth, the cross-sectional nature of the study limits causality-related conclusions. Seventh, while the finger pulse oximeter method used in the tasks has been validated against ECG for heartbeat timing, it does not provide the same level of temporal precision as ECG (17, 18). Finally, we investigated patients referred to a neurology clinic for their headaches, who by definition may represent a selective group with more disordered forms of migraine, compared to more common community-based individuals with migraine who do not require hospital referrals. Indeed, analysis of a broader spectrum of individuals would enable the assessment of whether those individuals with less disordered forms of migraines have better interoceptive accuracy. Therefore, the generalizability of our findings is limited, and further research is needed to address this issue.

## Conclusion

Although the cross-sectional nature of the study precludes causal inference, interoceptive abnormalities may represent transdiagnostic mechanisms relevant to the chronification of migraine, and also to frequently occurring co-morbidities such as dissociation. In the future, interoception could represent a novel transdiagnostic biomarker and therapeutic target for patients with migraine.

## Data Availability

The raw data supporting the conclusions of this article will be made available by the authors, without undue reservation.
